# Draft genome sequencing of *Enterococcus avium* strains isolated from bovine mastitis

**DOI:** 10.1128/mra.00236-24

**Published:** 2024-05-03

**Authors:** Monira Rahaman, Taniya Sultana, Md. Morshedur Rahman, Naim Siddique, Golam Mahbub Faisal, Mehedi Mahmudul Hasan, Ziban Chandra Das, A. N. M. Aminoor Rahman, Tofazzal Islam, M. Nazmul Hoque

**Affiliations:** 1 Molecular Biology and Bioinformatics Laboratory, Department of Gynecology, Obstetrics, and Reproductive Health, Bangabandhu Sheikh Mujibur Rahman Agricultural University (BSMRAU), Gazipur, Bangladesh; 2 Department of Fisheries and Marine Science, Noakhali Science and Technology University, Noakhali, Bangladesh; 3 Institute of Biotechnology and Genetic Engineering (IBGE), BSMRAU, Gazipur, Bangladesh; Montana State University, Bozeman, Montana, USA

**Keywords:** draft genomes, *Enterococcus avium*, multidrug resistant, bovine mastitis

## Abstract

We performed whole-genome sequencing of four multidrug-resistant *Enterococcus avium* strains isolated from milk (4M1), feces (4F1 and 4F2), and farm soil (4S1) of mastitic dairy cows. The draft genomes of *E. avium* strains 4M1, 4F1, 4F2, and 4S1 were approximately 4.2 Mbp, with 39.1% GC content and 66.5× coverage.

## ANNOUNCEMENT

Mastitis, a widespread and economically important disease in dairy animals, is caused by diverse microbes, including multidrug-resistant opportunistic pathogens (1, 2). Recent studies showed that microbiota from diverse sources can influence mastitis pathophysiology (3–5). *Enterococcus* spp., including *Enterococcus faecalis*, *Enterococcus faecium*, *Enterococcus avium*, and *Enterococcus durans*, primary mastitis-causing pathogens, with *E. faecalis* (approx. 80%) and *E. faecium* (approx. 10%–15%) being predominant (6, 7). Although various *Enterococcus* spp. have been identified from bovine mastitis (6, 7), data on the association of *E. avium* strains with mastitis pathophysiology are currently unavailable.

Milk, feces, and farm soil samples from cows with clinical mastitis were collected between August and September 2023 at a dairy farm located in the Gazipur district (24.09° N, 90.41° E) of Bangladesh. Milk samples were enriched on nutrient broth (Oxoid, UK) incubating overnight aerobically at 37°C. Subsequently, enriched samples were plated on nutrient agar (Oxoid, UK) for overnight incubation at 37°C, followed by streaking on Modified Edward medium (MEM) agar (Oxoid, UK) and incubation at 37°C for 24 h. One isolated colony per sample was then subcultured onto blood agar (Oxoid, UK) enriched with 5% sheep blood and incubated aerobically at 37 °C for 48 h. The plates were examined for their typical growth, morphological features, and hemolytic characteristics (8). Species-level identification of *E. avium* was performed through a VITEK-2 system v9.01 (9). Pure colonies from MEM agar were diluted in 0.45% saline to match a 0.5 McFarland standard and then inoculated into VITEK 2 system cards. After 3 h, the system identified four isolates (4M1, 4F1, 4F2, and 4S1) as *E. avium* based on their biochemical properties. These multidrug-resistant (resistant to nitrofurantoin, sulfonamides, nalidixic acid, oxacillin, doxycycline, tetracycline, imipenem, and aztreonam) isolates were subcultured in nutrient broth (Biolife, Italy) overnight at 37°C, followed by DNA extraction using the QIAamp DNA Mini Kit (QIAGEN, Germany). Libraries were created using Nextera DNA Flex Kit (Illumina, USA) from 1 ng DNA, followed by whole-genome sequencing on an Illumina MiSeq with a 2 × 250 protocol. Paired-end raw reads underwent trimming with Trimmomatic v0.39 (10) and quality checking through FastQC v0.11.7 (11). Reads with phred scores >20 (12) were assembled using SPAdes v3.15.5 (13). Genome annotation was performed using the NCBI Prokaryotic Genome Annotation Pipeline v6.6 (14) with the *Enterococcus* CheckM v1.2.2 marker set (15). For the prediction of antimicrobial-resistance genes (ARGs), virulence factor genes (VFGs), and metabolic functions in the draft genomes, ResFinder 4.0 (16), VFDB (17), and RAST server (18) were used, respectively. Default parameters were used for all software unless otherwise specified.

The draft genome size of four multidrug-resistant *E. avium* strains 4M1, 4F1, 4F2, and 4S1 is approximately 4.21 Mbp, with 98.37% genome completeness in CheckM. Genomes assembly and annotation statistics are given in Table 1. The final assembly of 4M1, 4F1, 4F2, and 4S1 genomes comprised 170, 165, 173, and 155 contigs, respectively, with 4,131, 4,130, 4,132, and 4,173 predicted coding sequences, respectively. One of the hallmark findings is the detection of 16 VFGs and 17 ARGs, present in all four genomes with a similarity of over 97.0%.

**TABLE 1:** General genomic features of the *E. avium* strains^*a*^

*E. avium* strains	Genomic features								
	Size (bp)	Coverage (×)	Gc (%)	Contigs	*N*_50_ (bp)	CDSs	Protein coding genes	Genome accession no.	SRA accession no.
4M1	4,213,172	66.5	39.1	170	44,465	4,131	4,083	JAXHVX000000000	SRR26980299
4F1	4,215,104	66.5	39.1	165	45,550	4,130	4,082	JAXHVW000000000	SRR26980298
4F2	4,212,140	66.5	39.1	173	43,365	4,132	4,086	JAXHVV000000000	SRR26980297
4S1	4,246,665	66.5	39.1	155	51,547	4,173	4,126	JAXHVY000000000	SRR26980300

^*a*^ Coding sequences.

## Data Availability

The draft genomes of *E. avium* strains 4M1, 4F1, 4F2, and 4S1 have been deposited at NCBI/GenBank under BioProject accession PRJNA1046255. Genome accessions are available in Table 1.

## AFTER EPUB

*[This article was published on 3 May 2024 with Monira Rahaman's surname spelled incorrectly. The spelling was corrected in the current version, posted on 9 May 2024.]*
